# Exploring the health-relevant poverty line: a study using the data of 663,000 individuals in Japan

**DOI:** 10.1186/s12939-019-1118-8

**Published:** 2019-12-30

**Authors:** Takashi Oshio

**Affiliations:** Institute of Economic Research, Hitotsubashi University, 2-1 Naka, Kunitachi, Tokyo 186-8603 Japan

**Keywords:** Poverty line, Poverty rate, Population health

## Abstract

**Background:**

Income poverty is known to be associated with poor health outcomes. However, the poverty line, which is used to calculate the poverty rate, is arbitrarily set without specific reference to health. This study explored the health-relevant poverty line to understand poverty in terms of population health.

**Methods:**

Using repeated cross-sectional data from approximately 663,000 individuals obtained from 11 waves of nationwide population surveys conducted in Japan from 1986 to 2016, we used two methods to calculate a health-relevant poverty line: (1) We searched for a poverty line that maximized the proportion-weighted relative underperformance in health among individuals whose income was below the poverty line (Method I). (2) We searched for a poverty line that maximized the likelihood of the logistic regression model to explain poverty in terms of health using a binary variable for below-the-poverty-line income (Method II). For both methods, we considered five health outcomes: Poor/fair and poor self-rated health, subjective symptoms, problems with daily life activities, and psychological distress, along with covariates.

**Results:**

Methods I and II indicated that the health-relevant poverty line should be drawn, respectively, at 72–86% and 67–69% of median income; this level is somewhat higher than the conventional 50% or 60%.

**Conclusions:**

The results suggest that there is a risk that the conventionally defined poverty line may underestimate poverty in terms of population health.

## Introduction

It is well-known that socioeconomic status is closely associated with health. Among other types of poverty, poverty in terms of income has been found to have an adverse impact on health [1–5]. Lower income tends to reduce one’s opportunity to purchase what is needed for good health, such as sufficient quantities of high-quality food and health care [6, 7]. Lower income is also associated with higher odds of behavioral risk factors including smoking, obesity, and physical inactivity [8–10]. In addition, other social risk factors mediate the association between income and health. For instance, low-income individuals tend to have lower educational attainment and less social capital and reside in less affluent neighborhoods [11, 12]. All these factors are likely to negatively impact health.

Hence, measuring the overall degree of income poverty is of great importance for assessing the extent to which a population’s health is exposed to income-related risks. At the same time, poor health can contribute to low income by limiting an individual’s ability to work and reducing economic opportunities [13]. This two-way causation between income and health also suggests the need for health-related measures to address the issue of income poverty in society as a whole. In addition, an increasing number of studies have investigated the association between income inequality in society as a whole and individual health [14–17], increasing the relevance of the poverty rate, which is closely related to income inequality, for public health.

However, the poverty rate is not entirely based on rigorous health considerations. When poverty is measured in relative terms—that is, defined as a lack of ability to access goods and services that are regarded as customary or the norm in any given country—the poverty line is usually considered to be 50% or 60% of the total population’s median household income [18, 19]. However, this threshold is more or less arbitrarily chosen and lacks any theoretical rationale. The same is true of the poverty rate, which is calculated based on the poverty line. Hence, we cannot rule out the possibility that the conventionally defined poverty rate may under or overestimate the degree of poverty in terms of health for society overall. To address income disparities in terms of health, both the poverty line and rate must be considered as they relate to health.

This study explored the “health-relevant” poverty line, which can roughly but effectively capture the degree of poverty in terms of a population’s health, using repeated cross-sectional data obtained from 11-wave population-based surveys conducted in Japan. We sought a poverty line that could most effectively divide the population into healthier individuals and less healthy ones. A poverty line that is too low may fail to capture individuals whose income is above the poverty threshold but are nevertheless as unhealthy as those below the line. A poverty line that is too high may also be ineffective in measuring population health because too many healthy individuals may fall below the line. Because there is no theory from which to derive the health-relevant poverty line, we employed two reasonable and easily operated statistical methods.

We utilized the data from Japan, a country, whose poverty rate is now in the highest tertile among OECD member nations [19] and found that the poverty rate in 2015 for income after taxes and transfers based on the 50% poverty line was 15.7%. In addition, an increasing proportion of non-regular employees points to the risk of higher exposure to lower income as well as unstable employment status [20]. Hence, the question of how health risks are linked to income poverty is now of great concern for the nation as a whole. Since our methods can be applied to all countries and societies, the current study’s findings are expected to provide new insight into income disparity in terms of health, a key issue to be addressed in public health policy.

## Methods

### Study sample

We utilized a dataset obtained from the Comprehensive Survey of Living Conditions (CSLC), a nationwide population-based survey conducted by the Japanese Government’s Ministry of Health, Labour and Welfare. The CSLC, conducted since 1986, comprises an annual household survey and a triennial health and income/savings survey. The CSLC samples are selected nationwide using a two-stage random sampling procedure. First, about 5400 districts are selected randomly from about 940,000 national census districts. Second, about 290,000 households are selected randomly from the selected district, according to population size. The response rate ranged from 77.6% (in 2016) to 95.7% (in 1986), and the valid sample rate (available only since 1995) ranged from 77.5% (in 2016) to 89.6% (in 1995). We used the data collected from each of the 11 waves that the CSLC conducted from 1986 to 2016 and restricted the study sample to individuals aged 15–89 years. After excluding respondents missing essential information, we utilized the data of 663,273 individuals (314,182 men and 349,091 women).

We obtained the CSLC data with permission from the MHLW. The CSLC was authorized by the Ministry of Internal Affairs and Communications, which is in charge of all government surveys in Japan, from the statistical, legal, ethical, and other viewpoints in accordance with the Statistics Law in Japan. Hence, ethics approval was not required for the current study.

### Measures

We focused on household income as an income variable. To adjust for household size, we divided the reported household income by the square root of the number of family members. This adjustment was based on recent publications by the OECD [21–23]. The income data were based on the income and tax records for one year prior to each survey year.

Five health outcomes were considered in this study: (i) poor/fair self-rated health (SRH), (ii) poor SRH, (iii) subjective symptoms, (iv) problems in the activities of daily life (ADL), and (v) psychological distress. Regarding SRH, the CSLC asked respondents, “What is your current health status? Is it excellent, very good, good, fair, or poor?” We constructed two types of binary variables for SRH: (i) 1 was allocated to fair or poor, while 0 was allocated to otherwise; or (ii) 1 was allocated to poor and 0 to otherwise.

As for subjective symptoms, the survey asked, “Have you been feeling ill due to sickness or injury for the past few days?” We constructed a binary variable for “having any subjective symptoms,” allocating 1 to those who answered “yes”; otherwise, 0 was allocated. Regarding ADL problems, the survey asked, “Do your health problems have any impact on the activities of your daily life?” We then constructed a binary variable for “having any ADL problems.” We allocated 1 to those who answered “yes”; otherwise, 0 was allocated.

To measure psychological distress, we employed Kessler 6 (K6) scores [24, 25]. First, we obtained the respondents’ assessments of psychological distress using a six-item psychological distress questionnaire—“During the past 30 days, approximately how often did you feel a) nervous, b) hopeless, c) restless or fidgety, d) so depressed that nothing could cheer you up, e) that everything required effort, and f) worthless?” Responses were rated on a 5-point scale (0 = *not at all* to 4 = *all of the time*). We then calculated the sum of the reported scores (range: 0–24) and defined it as the “K6 score,” with higher scores reflecting higher levels of psychological distress. The Cronbach’s alpha in this study sample was 0.963. We then constructed a binary variable for psychological distress to which we allocated a “1″ for K6 scores ≥13, which indicates serious mental disorder in the Japanese sample [26]. The SRH and subjective symptom data were available for the entirety of the 11 waves, while those for ADL problems and psychological distress were only available for 1989–2016 and 2007.

### Analytic strategy

We employed two methods (Methods I and II) to construct the health-relevant poverty line, based on the median income calculated in each survey year. Method I searched for a poverty line that maximized the proportion-weighted relative underperformance in health among individuals whose income was below the poverty line using the aggregated data. Method II searched for a poverty line that maximized the likelihood of the logistic regression model to explain poverty in terms of health using a binary variable for below-the-poverty-line income using micro data.

In Method I, we first drew the poverty line at *x* × 100% of median income, and then calculated *p*(*x*), the proportion of individuals in the entire population whose income was below this poverty line. We also calculated *m*(*x*), the proportion of those with the poor health outcome (such as poor SRH) among those below the poverty line. In the same way, we calculated the proportion of those with poor health outcomes in the entire population and denoted it as *m*. It was reasonable to predict that *m*(*x*) would be higher than *m*, assuming that health is positively related to income. We then searched for the value of *x* that maximized *p*(*x*)[*m*(*x*)–*m*], the proportion-weighted relative underperformance in health among individuals with below-the-poverty-line income, by gradually increasing the value of *x* from 0 at intervals of 0.01. We predicted that while a higher value of *x* would raise the proportion of individuals below the poverty line, it would reduce those individuals’ relative underperformance in health. We defined the poverty line corresponding to the value of *x* that maximized the product of these opposing effects as the most health-relevant poverty line.

In Method II, we first drew the poverty line at *x* × 100% of median income and constructed a binary variable of below-the-poverty-line income, and then for each *x* we estimated a logistic regression model for how well the binary variable explains the poorer health outcome for below-the-poverty-line income. We hypothesized that a higher value of the estimated odds ratio (OR) for the poorer health outcome for below-the-poverty-line income could indicate a higher relevance of the poverty line, but a higher standard error of the estimated OR would reduce the model’s reliability. To consider both factors, we focused on the likelihood (i.e., goodness of fit) of the regression model and defined the poverty line to correspond to the value of *x* that maximizes the (log-transformed) likelihood as the most health-relevant. We searched for it by gradually raising the value of *x* from zero, as with Method I.

In all regression models, we included a set of covariates for gender (female), 15 age groups (aged between 15 and 19 and 85–89 years), and 11 survey waves (1986–2016), all of which were constructed as binary variables. Because the number of explanatory variables was the same for all regression models, the assessment based on the likelihood was equivalent to that based on the Akaike information criteria. To assess the robustness of the health-relevant poverty lines derived from the entire population, for both Methods I and II we compared the results between men and women as well as the young (aged 15–64 years) and old (65–89). For the statistical analysis, we used the Stata software package (Release 15).

## Results

Table 1 presents the basic sample structure. For the entire sample, the poverty rate was 14.3 and 20.1% for the 50 and 60% poverty lines, respectively. One-third of the respondents reported subjective symptoms, slightly more than 10% reported poor/fair SRH and ADL problems, and less than 5% reported poor SRH and psychological distress. Table 2 summarizes the evolution of median income, the poverty lines, and poverty rates during the 11 waves. Over 20 years, the poverty rates fell in the range of 12.0 and 16.1% (for the 50% poverty line) and 17.9 and 22.0% (for the 60% poverty line).

**Table 1:** Key features of the study sample

		All	Men	Women
*N* in each survey year	1986	76,927	36,573	40,354
	1989	90,643	43,174	47,469
	1992	84,429	39,907	44,522
	1995	74,605	35,462	39,143
	1998	65,910	31,391	34,519
	2001	65,803	31,066	34,737
	2004	43,095	20,321	22,774
	2007	42,127	19,941	22,186
	2010	40,225	18,940	21,285
	2013	42,481	19,906	22,575
	2016	37,028	17,501	19,527
	Total	663,273	314,182	349,091
Age (years)	*M*	48.1	47.3	48.8
	*SD*	(18.8)	(18.4)	(19.0)
Proposition (%)				
Poverty rate (for 50% poverty line)		14.3	12.8	15.6
Poverty rate (for 60% poverty line)		20.1	18.4	21.7
Poor/fair SRH		13.0	12.2	13.8
Poor SRH		1.5	1.5	1.5
Subjective symptom		33.6	30.8	36.1
ADL problem (1989–2016)		11.8	11.3	12.3
Psychological distress (2007–2016)		4.0	3.5	4.5

Note. ^a^self-rated health. ^b^activities of daily life

**Table 2:** Conventionally defined poverty lines and poverty rates: 1986–2016

Survey year^a^	Median income	Poverty line	Poverty rate	Poverty line	Poverty rate
	(annual, million JPY)	(0.50 × median income)	(%)	(0.60 × median income)	(%)
1986	2.15	1.08	12.0	1.29	17.9
1989	2.27	1.14	13.2	1.36	19.5
1992	2.70	1.35	13.4	1.62	19.8
1995	2.89	1.44	13.8	1.73	19.9
1998	2.97	1.49	14.6	1.78	20.6
2001	2.72	1.36	15.4	1.63	21.5
2004	2.60	1.30	14.9	1.56	20.8
2007	2.53	1.27	15.7	1.52	21.7
2010	2.49	1.25	16.0	1.50	22.0
2013	2.44	1.22	16.1	1.46	21.9
2016	2.44	1.22	15.6	1.47	21.6

^a^Income data were reported one year prior to survey years

**Fig. 1:**
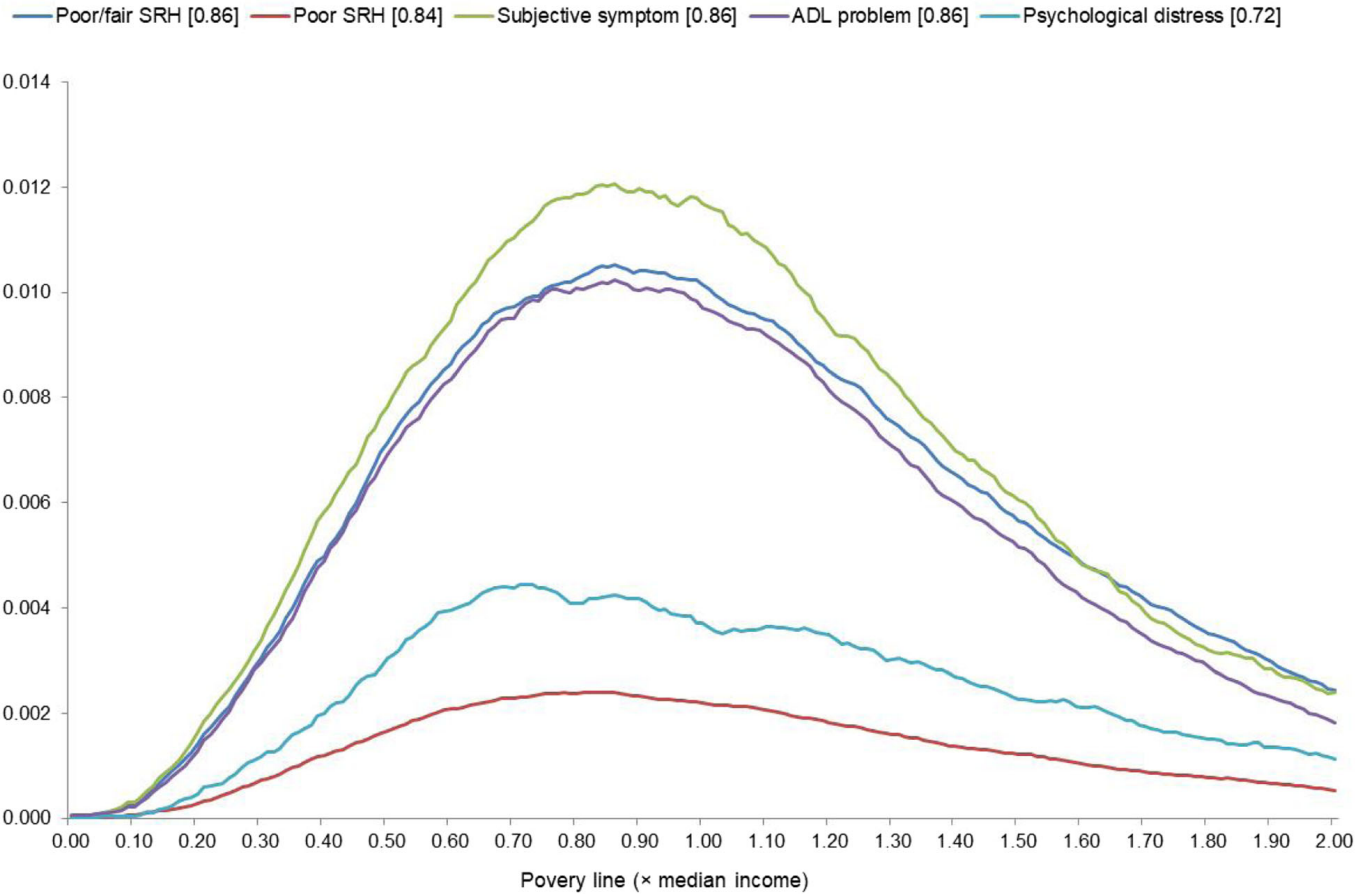
Proportion-weighted relative underperformance in health among individuals with below-the-poverty-line income (*p*(*x*)[*m*(*x*) – *m*])^a^. Note. ^a^ The figures in the brackets in the legend indicate the value of *x* that maximizes *p*(*x*)[*m*(*x*)–*m*]

To illustrate the results of Method I, Fig. 1 presents how the proportion-weighted relative underperformance in health among individuals with below-the-poverty-line income (*p*(*x*)[*m*(*x*)–*m*]) corresponded to the poverty line (*x*) for each health outcome. Each curve has a single peak—while the curve for psychological distress displayed some minor peaks to the right of the apex—meaning that each health outcome had a single, most health-relevant poverty line. In the legends, the figures in brackets indicate the value of *x* that maximizes *p*(*x*)[*m*(*x*)–*m*], showing that the most health-relevant poverty line corresponded to 72–86% of median income. Poor SRH and psychological distress have flatter curves than the others because, as seen in Table 1, these two outcomes had lower prevalence.

**Fig. 2:**
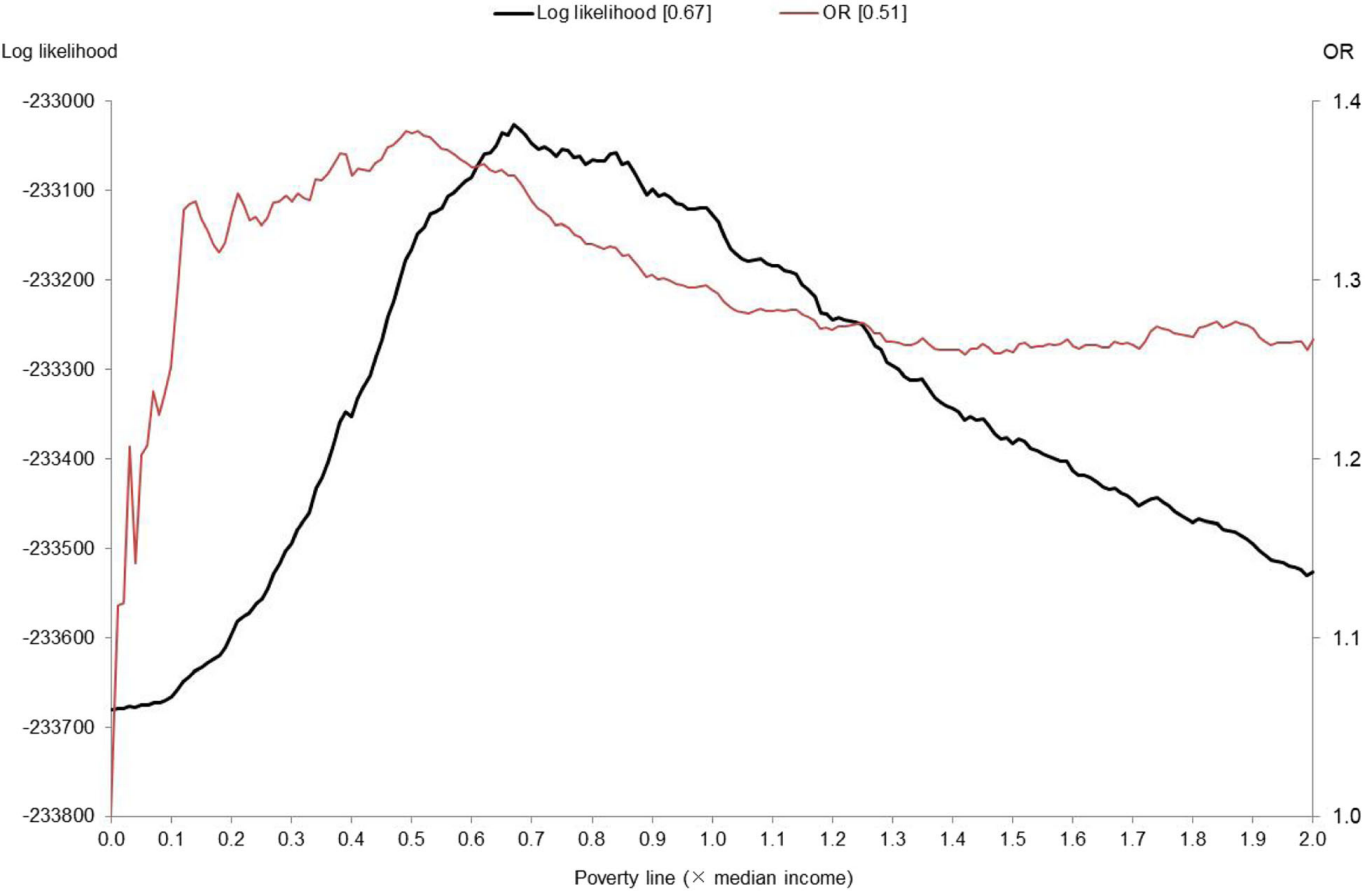
Log likelihood and odds ratio (OR) of poor/fair SRH^a^. Note. ^a^ self-rated health. The figures in the brackets in the legends indicate the values of *x* that maximize each

As an example of Method II, Fig. 2 shows how the log likelihood and OR of the poor health outcome corresponded to the poverty line in the case of poor/fair SRH. With an increase in *x* from zero, the OR rose over 1.3 when *x* = 0.1; peaked at 1.38 when *x* = 0.51; and, thereafter, declined gradually until *x* reached around 1.5. It is noteworthy that a very low poverty line (*x* < 0.1) led to a relatively low OR; this was probably because such a poverty line failed to capture poor-health individuals whose income was above the poverty line but still relatively low. A rise in the poverty line for *x* > 1.5 made the OR curve turn around, but the limited upturn suggests no substantial difference in health between individuals below and above the poverty line.

The log likelihood curve also showed clearer kurtosis with a peak at *x* = 0.67, which was somewhat higher than where the OR curve peaked at 0.51. At this peak, the OR was equal to 1.36, slightly lower than its peak of 1.38. This means that the regression model’s goodness of fit could be increased by raising the poverty line—that is, increasing the proportion of individuals categorized as poor—with only a modest reduction in the OR of the poor health outcome. We could depict similar figures for the other health outcomes as well; the log-likelihood curves peaked at a somewhat higher poverty line (with a higher value of *x*) than those of the ORs, and did not show a substantial decline in the OR from its peak.

Table 3 compares the health-relevant poverty lines obtained using these two methods as well as the poverty rates and prevalence of the poor health outcome—that is, the proportion of individuals who reported poor health outcome—below and above the poverty line. For reference, this table includes the results for the two types of conventionally defined poverty lines (*x* = 0.5 and 0.6). The health-relevant poverty lines obtained by Methods I and II corresponded, respectively, to 72–86% and 67–69% of median income, somewhat higher than the conventional figure of 50% or 60%. Accordingly, the proportion of individuals whose income was below the poverty line was 28–38% and 24–31% for Methods I and II, respectively, exceeding the levels corresponding to the conventionally defined poverty lines (around 14% for *x* = 0.50 and 20% for *x* = 0.60). We also observed that for all health outcomes the poverty lines obtained in Model I were somewhat higher than those in Model II, leading to higher poverty rates.

**Table 3:** Estimated poverty lines, poverty rates, and prevalence of poor health outcome

Health outcome	Poverty line		Poverty rate (%)	Prevalence (%) of poor health outcome		
		(× median income^a^)		Below the	Above the	Difference^d^
				poverty line (*A*)	poverty line (*B*)	(*A* – *B*)
Poor/fair SRH^b^	Conventionally defined	0.50	14.1	18.1	12.2	5.9
(*N* = 633,258)		0.60	19.9	17.4	11.9	5.4
	Method I	0.86	38.0	15.8	11.3	4.5
	Method II	0.67	24.4	17.0	11.8	5.2
Poor SRH	Conventionally defined	0.50	14.1	2.7	1.3	1.4
(*N* = 633,258)		0.60	19.9	2.5	1.2	1.3
	Method I	0.84	36.5	2.2	1.1	1.1
	Method II	0.68	25.1	2.4	1.1	1.3
Subjective symptom	Conventionally defined	0.50	14.3	39.1	32.7	6.4
(*N* = 633,258)		0.60	20.1	38.3	32.4	5.9
	Method I	0.86	38.3	36.7	31.6	5.1
	Method II	0.76	30.9	37.4	31.9	5.5
ADL^c^ problem	Conventionally defined	0.50	14.3	16.7	11.0	5.7
(*N* = 559,269)		0.60	20.1	16.0	10.8	5.2
	Method I	0.86	38.0	14.5	10.2	4.3
	Method II	0.68	25.2	15.6	10.5	5.0
Psychological distress	Conventionally defined	0.50	14.9	6.1	3.7	2.4
(*N* = 139,132)		0.60	20.5	5.9	3.5	2.4
	Method I	0.72	28.1	5.6	3.4	2.2
	Method II	0.69	26.1	5.7	3.4	2.3

Note. ^a^calculated in each year. ^b^self-rated health. ^c^activities of daily life. ^d^the maximum values for each health outcome are underlined

For each of the five health outcomes, below (*A*) and above (*B*) the poverty line, Table 3 also demonstrates the prevalence difference’s (*A* – *B*) sensitivity for poor health outcomes. For instance, the prevalence of poor/fair SRH was 15.8 and 11.3% below and above the poverty line in Method I. The difference in prevalence, 4.5 percentage points, was smaller than the 5.9 or 5.4 percentage points given for the conventionally defined poverty line. However, the reduction was relatively small compared to the increase in the poverty rate (from 14.1% or 19.9 to 38.0%). This is even truer of Method II, in which the poverty rate rose only to 24.4% and the difference in prevalence only declined to 5.2%. We obtained almost the same results for the other health outcomes.

Finally, we calculated the health-relevant poverty rates separately for men and women as well as the young (aged 15–64 years) and old (65–89). Table 4 summarizes the results. We found no substantial differences between men and women except for psychological distress, for which the poverty line was somewhat higher among men. Among the old, the poverty line was somewhat higher except for psychological distress, which revealed the opposite pattern. However, these differences were modest in all cases, underscoring the robustness of the health-relevant poverty line obtained from the entire sample. We also found that the poverty lines obtained in Model I were somewhat higher than those in Model II for all categories except old individuals, repeating the similar pattern observed for the entire sample reported in Table 3.

**Table 4:** Estimated health-relevant poverty lines by demographic group

Health outcome	Category	Method I	Method II
		*ratio to median income*^*a*^	
Poor/fair SRH	All	0.86	0.67
(*N* = 633,258)	Men	0.84	0.67
	Women	0.86	0.68
	Young^d^	0.83	0.67
	Old^e^	0.84	0.84
Poor SRH^b^	All	0.84	0.68
(*N* = 633,258)	Men	0.84	0.67
	Women	0.86	0.68
	Young	0.68	0.65
	Old	0.76	0.76
Subjective symptom	All	0.86	0.76
(*N* = 633,258)	Men	0.84	0.78
	Women	0.86	0.76
	Young	0.77	0.76
	Old	0.84	0.99
ADL^c^ problem	All	0.86	0.68
(*N* = 559,269)	Men	0.84	0.67
	Women	0.86	0.68
	Young	0.75	0.66
	Old	0.76	0.76
Psychological distress	All	0.72	0.69
(*N* = 139,132)	Men	0.73	0.72
	Women	0.68	0.69
	Young	0.86	0.73
	Old	0.68	0.68

Note. ^a^calculated in each year. ^b^self-rated health. ^c^activities of daily life. ^d^aged 15–64 years. ^e^aged 65–89 years

## Discussion

This study explored the health-relevant poverty line using two methods and a large dataset obtained from nationwide population surveys in Japan. To estimate the health-relevant poverty line, we explored two methods. Using aggregated data, Method I searched for a poverty line that maximized the proportion-weighted relative underperformance in health among individuals with below-the-poverty-line income. Method II used microdata to search for a poverty line that maximized the likelihood of the logistic regression model to explain poverty in terms of health using a binary variable of below-the-poverty-line income. The key findings are summarized as follows.

First, the results showed that the health-relevant poverty line should be somewhat higher than the conventionally defined line. Specifically, Methods I and II showed that the health-relevant poverty line should be drawn, respectively, at 72–86% and 67–69% of median income, higher than the conventional definition of 50% or 60%. Correspondingly, the poverty rate calculated using the health-relevant poverty line exceeded the conventional one. For the poverty rates, Methods I and II obtained 28–38% and 24–31%, respectively, exceeding the poverty levels that correspond to the conventionally defined poverty line.

Second, raising the poverty line from the conventionally defined line to the health-relevant one could increase the poverty rate without a substantial reduction in the relative underperformance in health among individuals who are living below the poverty line. In general, there may be a trade-off between the poverty line and relative underperformance in health; as the poverty line rises, the poverty rate rises while relative underperformance in health declines. However, we observed that the relative underperformance in health was not highly sensitive to a rise in the poverty line unless the health-relevant poverty line was too far from the conventional one.

Third, the health-relevant poverty line obtained for the entire population was largely applicable when the population was divided into men/women and young/old. For each demographic group, we obtained a result for the health-relevant poverty line that was not far from that of the entire population.

Lastly, looking more closely at the results, we found that the health-relevant poverty lines obtained in Model I tended to be somewhat higher than those obtained in Model II. A possible reason for this difference between the two models can be explained as follows. In Model II, a rise in the poverty line from a sufficiently high level will directly reduce the likelihood of the model. In Model I, by contrast, a rise in the poverty line from a sufficiently high level will not much reduce the relative underperformance in health among the below-the-poverty-line individuals (*m*(*x*)–*m*). This effect, which does not hold in Model II, is likely to make the proportion-weighted value (*p*(*x*)[*m*(*x*)–*m*]) peak at a higher *x* than in Model II. It should be noted, however, that the health-relevant poverty lines were not much different between the two models, although the results in Model I, which used the aggregated data, may not be free from the effects of the ecological fallacy. This is probably because both models captured a trade-off between the poverty line and relative underperformance in health among the below-the-poverty-line individuals.

Combined, these results suggest that the health-relevant poverty line should be drawn at a level somewhat higher than the conventionally defined poverty line. In other words, the results point to a risk that the conventional poverty line may underestimate the degree of poverty in terms of population health. Considering that we obtained very similar health-relevant poverty lines for men/women as well as young/old, we can also argue that a single health-relevant poverty line should be used to capture the extent of poverty in the population’s overall health.

We acknowledge that the current study had several limitations. First, we should be cautious about any generalization of this study’s observations, because all health outcomes used were self-reported and because we only used data from the Japanese population. Self-reported outcomes are not free from measurement errors and are likely to be affected by individual attributes, both observed and unobserved. In addition, the estimated level of the health-relevant poverty line and its comparison with conventional poverty lines must differ from country to country as a result of different national socioeconomic and institutional backgrounds.

Second, we addressed poverty in relative terms, implicitly assuming that lack of an ability to access the goods and services that are regarded as customary or the norm in any given country may cause health to deteriorate. This “relative poverty” approach is consistent with the view that health is associated with relative income deprivation [27, 28]. Indeed, one study has even demonstrated that mortality has a closer association with relative than absolute poverty [29]. However, the “absolute poverty” approach, which focuses on an absolute minimum income threshold necessary for survival, also may be useful, especially in developing countries. Even in advanced countries, another approach can be applied; for instance, the poverty line can be semi-normatively set at the household income level at which a healthy standard of living can be realistically achieved through diet, nutrition, and other factors [30]. These approaches must be complementary to the relative poverty approach taken in this study.

Third, we focused exclusively on poverty in terms of income, ignoring other aspects. Health has been found to be associated with multidimensional poverty, which incorporates deprivations in not only income but also other aspects of life such as educational background, residential conditions, and safety nets [31, 32]. In addition, future research should consider the possible confounding effects on the association between income poverty and health, as already pointed out by preceding studies [33–35]. In addition, future research should consider possible confounding effects on the association between income poverty and health, as already pointed out in preceding studies [33–35].

## Conclusions

We presented two methods for constructing the health-relevant poverty line. Both methods suggest that the health-relevant poverty line in Japan should be drawn at a level somewhat higher than the conventional poverty line. We believe that this study’s observations provide new insights into income disparities in terms of population health. Furthermore, the two methods presented here point to a need for further research to elucidate the degree of poverty in terms of income as well as other socioeconomic factors with specific reference to health.

## Data Availability

The data that support the findings of this study are available from the MHLW but restrictions apply to the availability of these data, which were used under licence for the current study and so are not publicly available. Data are, however, available from the authors upon reasonable request and with permission of the MHLW.

## Abbreviations

ADL: Activities of daily life
CSLC: Comprehensive Survey of Living Conditions
K6: Kessler 6
MHLW: Ministry of Health, Labour and Welfare
OECD: Organisation for Economic Co-operation and Development
OR: Odds ratio
SRH: Self-rated health

## References

[1] Lorant V, Deliege D, Eaton W, Robert A, Philippot P, Ansseau M (2003). Socioeconomic inequalities in depression: a meta-analysis. Am J Epidemiol.

[2] Mackenbach JP, Stirbu I, Roskam AJ, Schaap MM, Menvielle G, Leinsalu M (2008). Socioeconomic inequalities in health in 22 European countries. N Engl J Med.

[3] Marmot M (2005). Social determinants of health inequalities. Lancet.

[4] World Health Organization. Poverty and health. 2019. https://www.who.int/hdp/poverty/en/

[5] Zimmerman FJ, Katon W (2005). Socioeconomic status, depression disparities, and financial strain: what lies behind the income-depression relationship?. Health Econ.

[6] Bhattacharya J, Currie J, Haider S (2004). Poverty, food insecurity, and nutritional outcomes in children and adults. J Health Econ.

[7] Morris S, Sutton M, Gravelle H (2005). Inequity and inequality in the use of health care in England: an empirical investigation. Soc Sci Med.

[8] Jarvandi S, Yan Y, Schootman M (2002). Income disparity and risk of death: the importance of health behaviors and other mediating factors. PLoS One.

[9] Prentice C, McKillop D, French D (2017). How financial strain affects health: evidence from the Dutch National Bank Household Survey. Soc Sci Med.

[10] Stringhini S, Sabia S, Shipley M, Brunner E, Nabi H, Kivimaki M (2010). Association of socioeconomic position with health behaviors and mortality. JAMA.

[11] Cattell V (2001). Poor people, poor places, and poor health: the mediating role of social networks and social capital. Soc Sci Med.

[12] Mood C, Jonsson JO (2016). The social consequences of poverty: an empirical test on longitudinal data. Soc Indic Res.

[13] Daepp MIG, Arcaya MC (2017). The effect of health on socioeconomic status: using instrumental variables to revisit a successful randomized controlled trial. Econ Hum Biol.

[14] Kennedy BP, Kawachi Glass IR, Prothrow-Stith D (1998). Income distribution, socioeconomic status, and self rated health in the United States: multilevel analysis. BMJ.

[15] Kondo N, Sembajwe G, Kawachi I, van Dam RM, Subramanian SV, Yamagata Z (2009). Income inequality, mortality, and self rated health: meta-analysis of multilevel studies. BMJ.

[16] Oshio T, Kobayashi M (2009). Income inequality, area-level poverty, perceived aversion to inequality, and self-rated health in Japan. Soc Sci Med.

[17] Subramanian SV, Kawachi I (2004). Income inequality and health: what have we learned so far?. Epidemiol Rev.

[18] Union E (2019). Glossary: at-risk-of-poverty rate.

[19] Organisation for Economic Co-operation and Development. Poverty rate. 2019. https://data.oecd.org/inequality/poverty-rate.htm. Accessed 21 Sept 2019.

[20] Sekine Y (2008). The rise of poverty in Japan: the emergence of the working poor. Japan Labor Review.

[21] Organisation for Economic Co-operation and Development (2009). Growing unequal?. Income distribution and poverty in OECD Countries.

[22] Organisation for Economic Co-operation and Development (2011). Divided we stand. Why inequality keeps rising.

[23] Organisation for Economic Co-operation and Development (2015). All on board. Making inclusive growth happen.

[24] Kessler RC, Andrews G, Colpe LJ, Hiripi E, Mroczek DK, Normand SL (2002). Short screening scales to monitor population coverages and trends in non-specific psychological distress. Psychol Med.

[25] Kessler RC, Green JG, Grube MJ, Sampson NA, Bromet E, Cuitan M (2010). Screening for serious mental illness in the general population with the K6 screening scale: results from the WHO world mental health (WMH) survey initiative. Int J Methods Psychiatric Res.

[26] Sakurai K, Nishi A, Kondo K, Yanagida K, Kawakami N (2011). Screening performance of K6/K10 and other screening instruments for mood and anxiety disorders in Japan. Psychiatry Clin Neurosci.

[27] Adjaye-Gbewonyo K, Kawachi I (2012). Use of the Yitzhaki index as a test of relative deprivation for health outcomes: a review of recent literature. Soc Sci Med.

[28] Subramanyam M, Kawachi I, Berkman L, Subramanian SV (2009). Relative deprivation in income and self-rated health in the United States. Soc Sci Med.

[29] Fritzell J, Rehnberg J, Bacchus Hertzman J, Blomgren J (2015). Absolute or relative? A comparative analysis of the relationship between poverty and mortality. Int J Public Health.

[30] Deeming C (2009). Determining semi-normative poverty lines using social survey data. Soc Policy Adm.

[31] Callander EJ, Schofield DJ, Shrestha RN (2013). Chronic health conditions and poverty: a cross-sectional study using a multidimensional poverty measure. BMJ Open.

[32] Oshio T, Kan M (2014). Multidimensional poverty and health: evidence from a nationwide survey in Japan. Int J Equity Health.

[33] Niedzwiedz CL, Richardson EA, Tunstall H, Shortt NK, Mitchell RJ, Pearce JR (2016). The relationship between wealth and loneliness among older people across Europe: is social participation protective?. Prev Med.

[34] Wang R, Feng Z, Liu Y, Lu Y. Relationship between neighbourhood social participation and depression among older adults: a longitudinal study in China. Health Soc Care Community. 2019; [Epub ahead of print]. 10.1111/hsc.12859 31595604

[35] Wang R, Liu Y, Xue D, Helbich M (2019). Depressive symptoms among Chinese residents: how are the natural, built, and social environments correlated?. BMC Public Health.

